# Heart Failure Phenotypes Induced by Knockdown of DAPIT in Zebrafish: A New Insight into Mechanism of Dilated Cardiomyopathy

**DOI:** 10.1038/s41598-017-17572-y

**Published:** 2017-12-12

**Authors:** Yoji Nagata, Masakazu Yamagishi, Tetsuo Konno, Chiaki Nakanishi, Yoshihiro Asano, Shin Ito, Yuri Nakajima, Osamu Seguchi, Noboru Fujino, Masa-aki Kawashiri, Seiji Takashima, Masafumi Kitakaze, Kenshi Hayashi

**Affiliations:** 1 Division of Cardiovascular Medicine, Kanazawa University Graduate School of Medicine, Kanazawa, Japan; 2 Department of Cardiovascular Medicine, Osaka University Graduate School of Medicine, Suita, Japan; 3 Department of Clinical Research and Development, National Cerebral and Cardiovascular Center, Suita, Japan; 4 Department of Cell Biology, National Cerebral and Cardiovascular Center, Suita, Japan; 5 Department of Transplantation, National Cerebral and Cardiovascular Center, Suita, Japan; 6 Department of Medical Biochemistry, Osaka University Graduate School of Medicine, Suita, Japan

## Abstract

The pathogenesis of heart failure associated with dilated cardiomyopathy (DCM) may result in part from adenosine triphosphate (ATP) dysregulation in the myocardium. Under these conditions, diabetes-associated protein in insulin-sensitive tissue (DAPIT), which is encoded by the upregulated during skeletal muscle growth 5 (*USMG5*) gene, plays a crucial role in energy production by mitochondrial ATP synthase. To determine whether USMG5 is related to the development of heart failure, we performed clinical and experimental studies. Microarray analysis showed that the expression levels of USMG5 were positively correlated with those of natriuretic peptide precursor A in the human failed myocardium. When endogenous z-usmg5 in zebrafish was disrupted using morpholino (MO) oligonucleotides, the pericardial sac and atrial areas were larger and ventricular fractional shortening was reduced compared to in the control MO group. The expression levels of natriuretic peptides were upregulated in the z-usmg5 MO group compared to in controls. Further, microarray analysis revealed that genes in the calcium signalling pathway were downregulated in the z-usmg5 MO group. These results demonstrate that DAPIT plays a crucial role in the development of heart failure associated with DCM and thus may be a therapeutic target for heart failure.

## Introduction

Heart failure is a leading cause of mortality, even in developed countries. The prevalence of heart failure among the elderly population (>70 years old) is approximately 10% and is estimated to be increasing^1,2^. Despite improvements in pharmacological and mechanical therapies, the prognosis of heart failure patients remains poor, with a 1-year mortality of 25–30%^3^. Therefore, dissecting the molecular mechanisms of heart failure progression is necessary to develop novel therapeutics.

Dilated cardiomyopathy (DCM) is an idiopathic cardiomyopathy condition causing progressive left ventricular enlargement and systolic dysfunction related to advanced heart failure and cardiac transplantation. Although gene mutations are evident in 30–35% of DCM patients^4,5^, the mechanism of disease progression remains unclear. As the heart consumes more oxygen than all other human organs, myocardial energy metabolism is an important factor affecting disease progression in DCM^5^. Furthermore, impaired adenosine triphosphate (ATP) utilization in the myocardium has been associated with the severity and mortality of DCM^6^.

Mitochondria are organelles that produce most of the cellular ATP in cardiomyocytes. In this organelle, ATP synthase is an important enzyme complex that generates ATP, functioning in the last step of the mitochondrial oxidative phosphorylation system^7,8^. Diabetes-associated protein in insulin-sensitive tissue (DAPIT) is encoded by the upregulated during skeletal muscle growth 5 (*USMG5*), which is a component of the F_o_ subunit of the ATP synthase^9^^,^^10^ and plays a crucial role in energy production during ATP synthase^11^. However, the role of DAPIT in the myocardium has not been investigated. In this study, we hypothesized that DAPIT, a component of ATP synthase, plays a crucial role in the pathogenesis of heart failure associated with DCM.

## Results

### Expression levels of USMG5 and natriuretic peptide precursor A in failed myocardium of NICM patients

**Figure 1:**
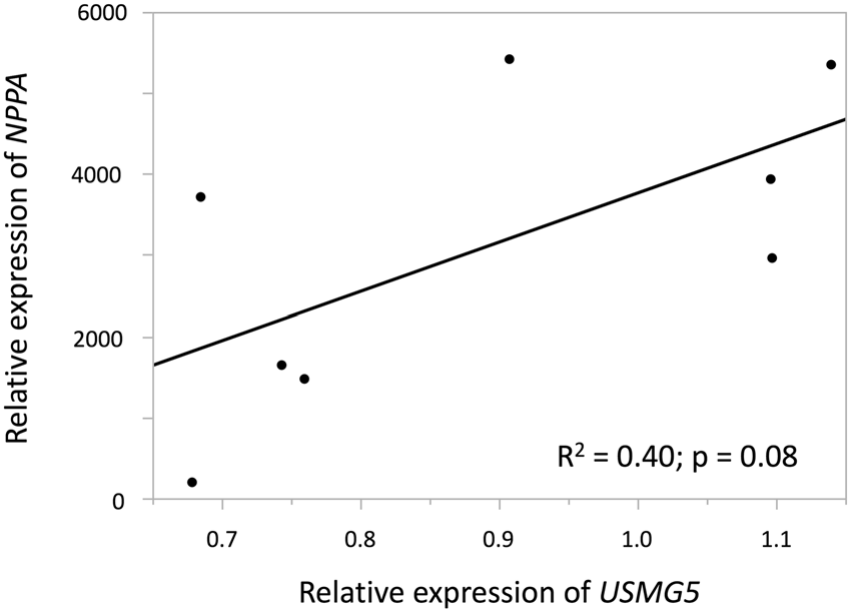
Microarray data revealed that the relative expression levels of *USMG5* were generally positively correlated with those of *NPPA* in the failed myocardium of NICM patients (R^2^ = 0.40, p = 0.08) in linear regression analysis. *NPPA*: natriuretic peptide precursor A, NICM: non-ischemic cardiomyopathy.

We first investigated whether the expression levels of *USMG5* are associated with the severity of heart failure in patients with non-ischemic cardiomyopathy (NICM) by microarray analysis. The relative expression levels of *USMG5* were generally positively correlated with those of natriuretic peptide precursor A *(NPPA)* in failed myocardium (R^2^ = 0.40, p = 0.08, Fig. 1). There were no significant differences in the relative expression levels of *NPPA* and *USMG5* between control subjects (n = 2) and NICM patients (n = 8) (Suppl. Figure S1)

### Phenotypes recapitulating heart failure induced by z-usmg5 MO injection in zebrafish embryos

**Figure 2:**
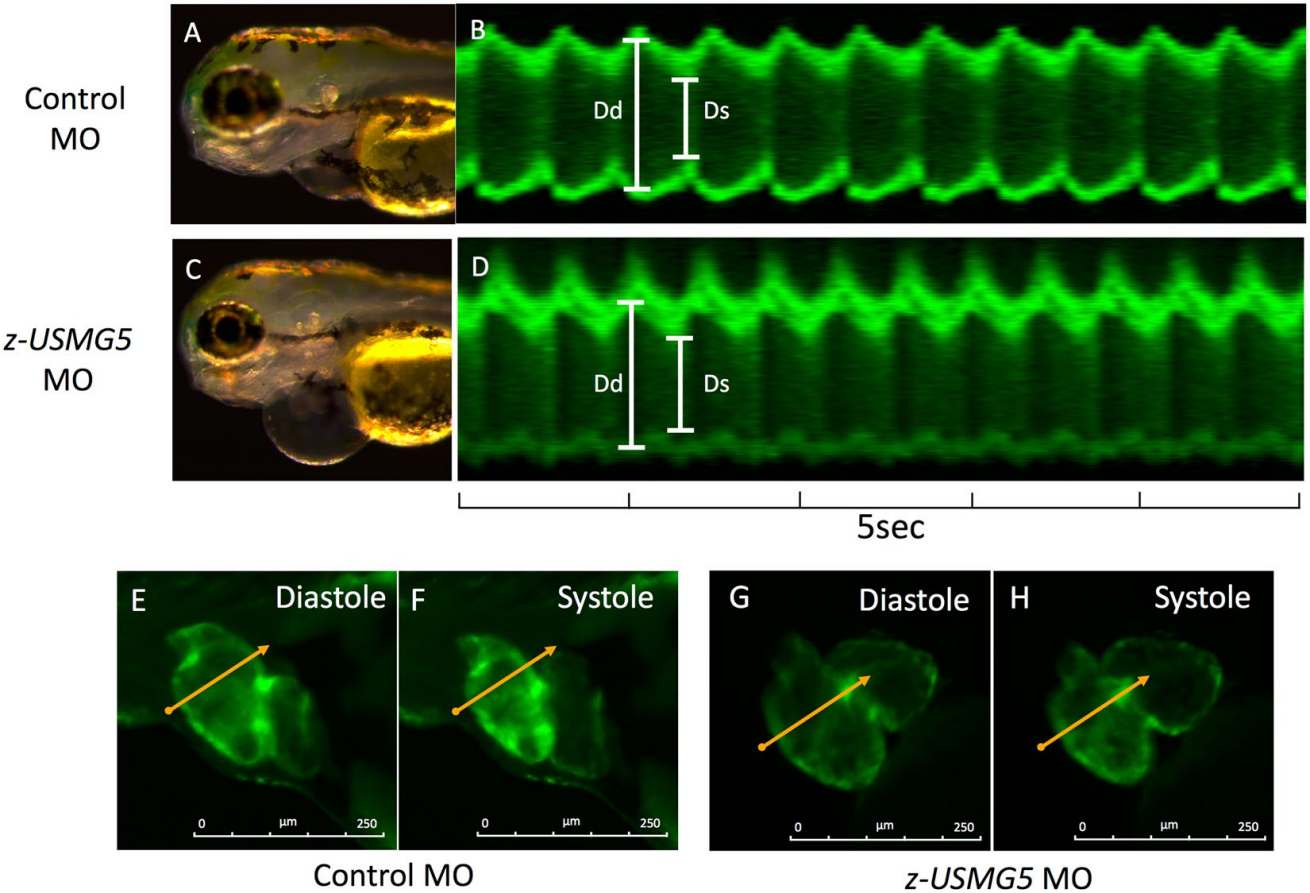
Representative images of zebrafish hearts at 72 hpf. Brightfield and fluorescent microscopy images of zebrafish embryos injected control MO (**A**,**B**) and *z-usmg5* MO (**C**,**D**), respectively. *z-usmg5* MO injected embryos showed swollen pericardial sacs (**C**) and reduced ventricular contraction (**D**,**G**,**H**) compared to the control MO embryos (**B**,**E**,**F**). hpf: hours post fertilization, MO: morpholino oligonucleotide, Dd: diastolic diameter, Ds: systolic diameter.

**Figure 3:**
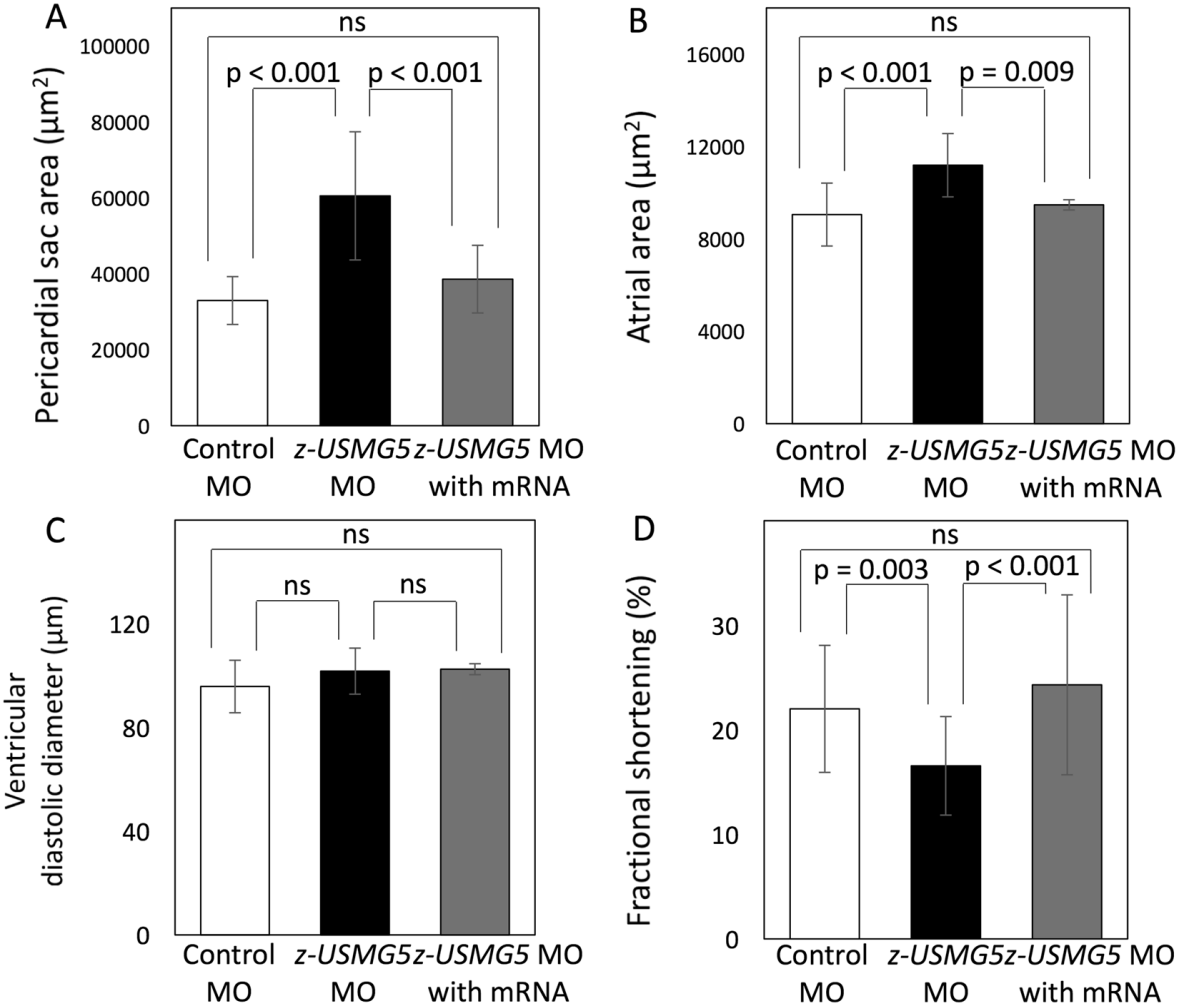
Quantitative measurements of cardiac dimensions and functions in the zebrafish embryos injected with control MO (control MO group, n = 21), *z-usmg5* MO (*z-usmg5* MO group, n = 19) and *z-usmg5* MO with its wild-type mRNA (*z-usmg5* MO with mRNA group, n = 20). Compared to the control, the MO group showed that the pericardial sac area (60,520 ± 16,872 μm^2^ vs. 32,962 ± 6,295 μm^2^, p < 0.0001) (**A**) and the atrial area (11,190 ± 1,370 μm^2^ vs. 9,052 ± 1,361 μm^2^, p < 0.0001) (**B**) were significantly increased. Although there were no differences in ventricular diastolic diameter (**C**), fractional shortening was significantly reduced in the *z-usmg5* MO group compared to in the control MO group (16.5 ± 4.7% vs. 22.0 ± 6.0%, p = 0.003) (**D**). These heart failure phenotypes caused by *z-usmg5* knockdown were rescued by co-injection of its wild-type mRNA ((**A**–**D**), *z-usmg5* MO with mRNA group). Statistical analyses were performed using Mann-Whitney U test.

We then examined whether knockdown of *z-usmg5* induced phenotypes of heart failure in zebrafish embryos. Compared to control MO-injected embryos, the percentage of embryos with a swollen pericardial sac (>2 SD from wild-type embryos) was significantly greater in z-usmg5 MO-injected embryos at both 48 and 72 hours post-fertilization (hpf) (Suppl. Figure S2). Evaluation at 72 hpf revealed no differences in heart rates between control MO (161 ± 12 bpm), *z-usmg5* MO (158 ± 14 bpm), and *z-usmg5* MO with its wild-type mRNA (152 ± 12 bpm) groups (Suppl. Figure S3). However, the *z-usmg5* MO group (n = 19) showed a larger pericardial sac area (60,520 ± 16,872 μm^2^ vs. 32,962 ± 6,295 μm^2^, p < 0.0001) and atrial area (11,190 ± 1,370 μm^2^ vs. 9,052 ± 1,361 μm^2^, p < 0.0001) than the control MO group (n = 21) (Figs 2 and 3A,B). Further, ventricular fractional shortening was significantly reduced in the *z-usmg5* MO group compared to in the control MO group (16.5 ± 4.7% vs. 22.0 ± 6.0%, p = 0.003) (Figs 2 and 3D). Importantly, there were no differences in cardiac morphology and function, such as pericardial area, atrial area, ventricular diameter, and ventricular fractional shortening between the wild-type embryos and control MO group (Suppl. Table S1).

To confirm that the observed phenotypes were specific to *z-usmg5* deficiency, we conducted rescue experiments. Co-injection of wild-type *z-usmg5* mRNA with its MO (n = 20) resulted in a significant reduction in the pericardial sac and atrial areas and improvement in ventricular fractional shortening compared to injection of only *z-usmg5* MO (Fig. 3A−D).

### Changes in foetal cardiac genes and ATP synthase-related genes in z-usmg5 MO-injected zebrafish embryos

**Figure 4:**
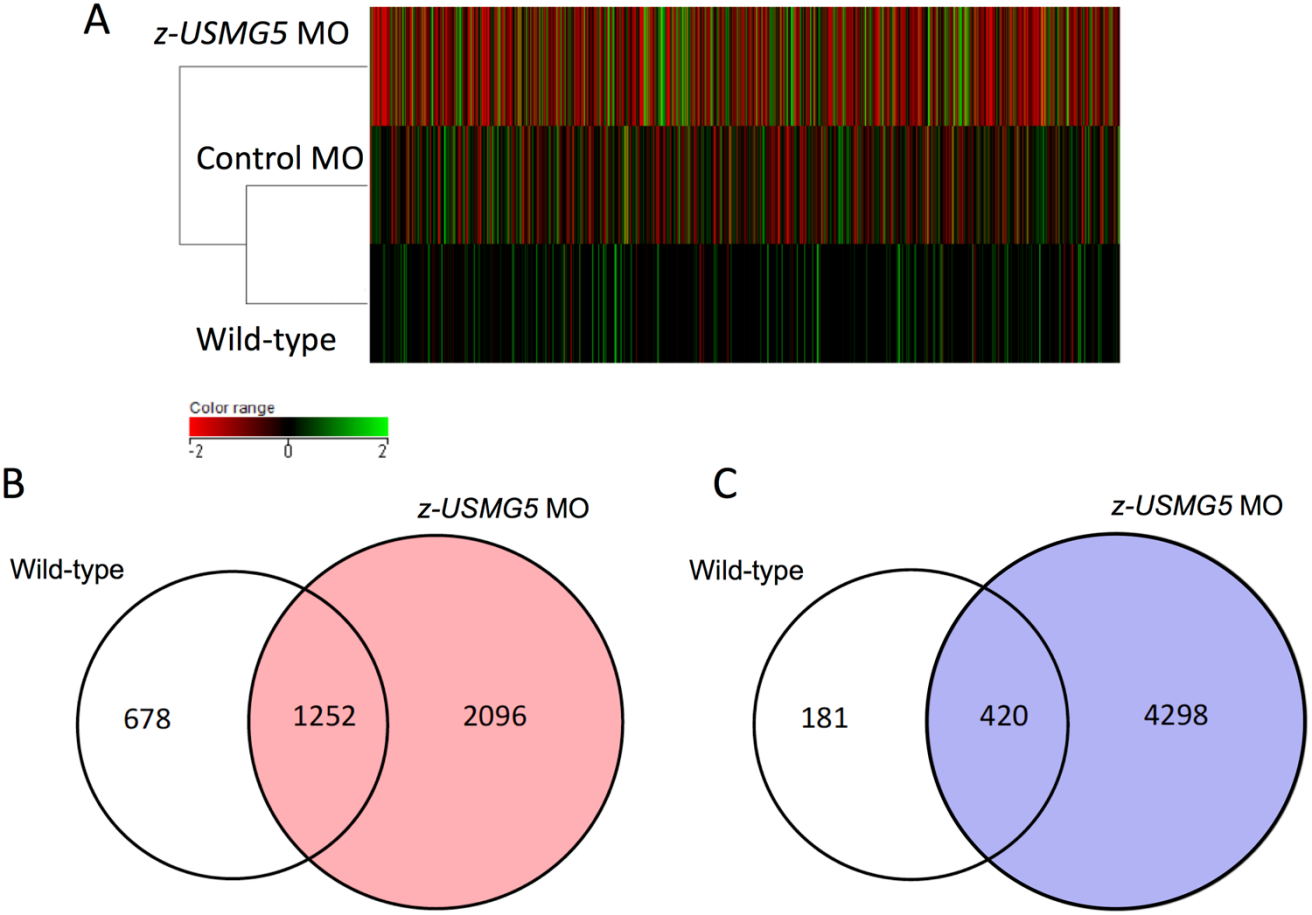
Hierarchical clustering analysis revealed that differences in gene expression profiles were more evident between wild-type embryos and *z-usmg5* MO embryos than between wild-type embryos and control MO embryos (**A**). A Venn diagram showing that 2,096 genes were upregulated > 1.5-fold (**B**) and 4,298 genes were downregulated by > 1.5-fold (**C**) in *z-usmg5* MO embryos compared to wild-type embryos.

We next performed microarray analysis to examine the differences in gene expression between *z-usmg5* MO-injected, control MO-injected, and wild-type embryos. Hierarchical clustering analysis revealed that differences in gene expression profiles were more evident between wild-type embryos and *z-usmg5* MO embryos than between wild-type embryos and control MO embryos (Fig. 4A). Compared to that in wild-type, 2,096 genes were upregulated (Fig. 4B) and 4,298 genes were downregulated (Fig. 4C) in *z-usmg5* MO embryos when a fold-change of 1.5 was set as the minimum.

Gene Ontology (GO) analysis revealed that 58 GO terms were upregulated and 133 were downregulated in *z-usmg5* MO embryos compared to in wild-type embryos. Importantly, GO terms related to ATP driving intracellular ions transfer, such as ATPase activity coupled to the transmembrane movement of ions (GO: 0042625), substrate-specific transmembrane transporter activity (GO: 0022891), and ion transmembrane transporter activity (GO: 0015075), were significantly downregulated (Table 1).

**Table 1:** Gene ontology analysis revealed that intracellular ATP depletion occurred in *z-usmg5* MO-injected zebrafish embryos compared to wild-type embryos.

GO ACCESSION	GO Term	p-value
GO:0051234	establishment of localization	<0.0001
GO:0005215|GO:0005478	transporter activity	<0.0001
GO:0006811	ion transport	<0.0001
GO:0022892	substrate-specific transporter activity	<0.0001
GO:0015075	ion transmembrane transporter activity	<0.0001
GO:0022891	substrate-specific transmembrane transporter activity	<0.0001
GO:0042625	ATPase activity, coupled to transmembrane movement of ions	0.0002

**Figure 5:**
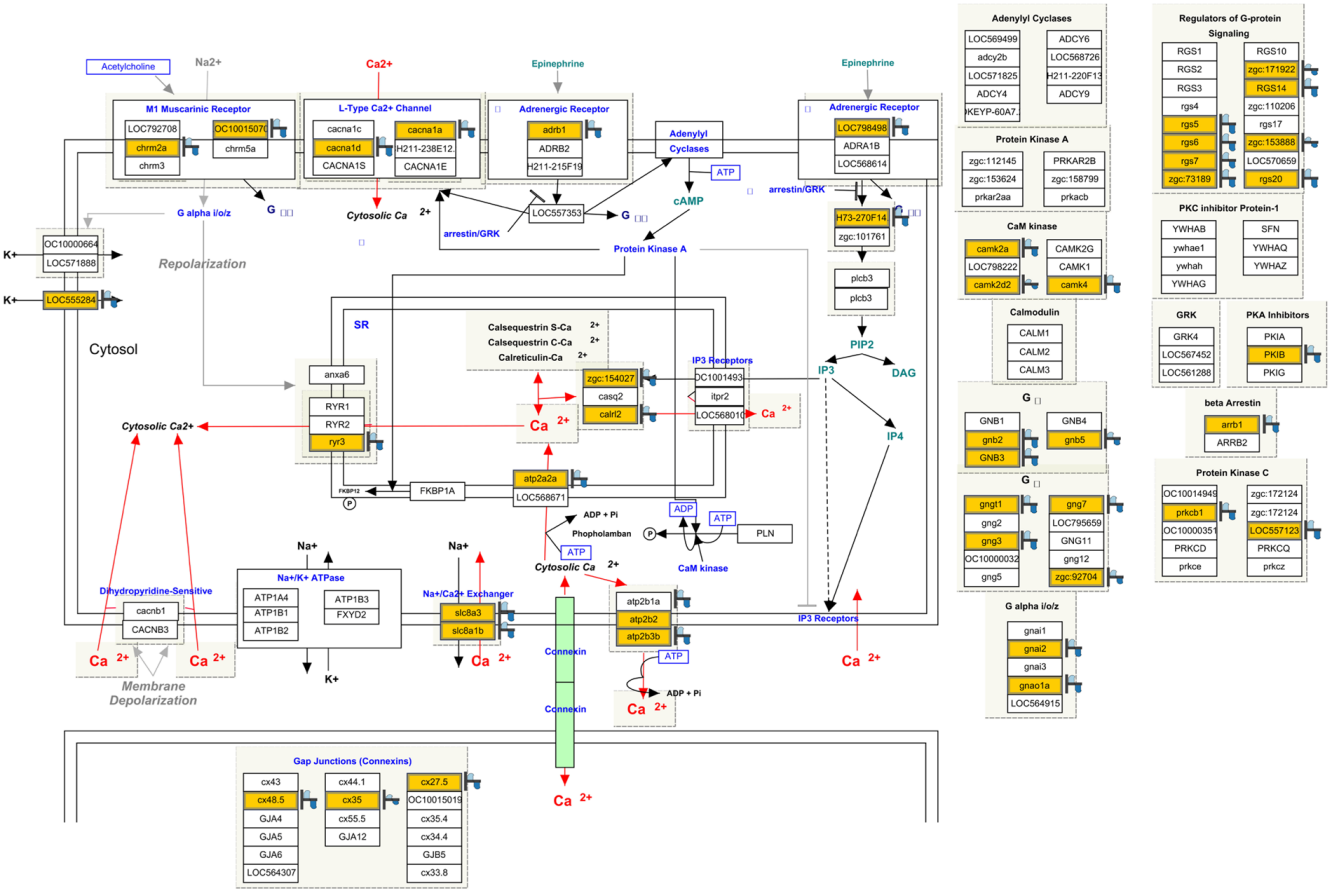
Pathway analysis using WikiPathways revealed that the factors related to the calcium signalling pathway (Kelder, T., Chichester, C., Hanspers, K.: Calcium Regulation in the Cardiac Cell (Danio rerio). http://www.wikipathways.org/instance/WP1365_r71502) were significantly downregulated (p < 0.0001) in *z-usmg5* MO embryos compared to in wild-type embryos. Downregulated genes in *z-usmg5* MO embryos with a fold change > 1.5 compared to wild-type embryos are highlighted in yellow boxes.

Pathway enrichment analysis revealed that 7 pathways were downregulated and 11 pathways were upregulated in embryos injected with *z-usmg5* MO compared to in wild-type embryos (Table 2). Among the 7 downregulated pathways, major factors involved in calcium signalling, such as *atp2a2* encoding sarcoplasmic/endoplasmic reticulum Ca^2+^-ATPase (SERCA), *slc8a1b* encoding a member of proteins constituting the sodium/calcium exchanger (NCX), *ryr3* encoding a ryanodine receptor (RyR), and *atp2b3* encoding a plasma membrane calcium ATPase, were significantly downregulated (p < 0.0001) in *z-usmg5* MO embryos compared to in wild-type embryos (http://www.wikipathways.org/index.php/Pathway:WP1365; Fig. 5). Furthermore, the transforming growth factor β signalling pathway was significantly upregulated (p < 0.0001) in *z-usmg5* MO embryos compared in wild-type embryos.

**Table 2:** Pathway enrichment analysis using WikiPathways revealed significantly activated pathways in *z-usmg5* MO-injected zebrafish embryos compared to wild-type embryos.

Pathways	p-value	Matched Entities	Pathway Entities
*Significantly activated pathways in downregulated genes*			
Dr_Calcium_Regulation_in_the_Cardiac_Cell_WP1365_71502	<0.0001	43	110
Dr_GPCRs_Class_C_Metabotropic_glutamate_pheromone_WP1373_68623	<0.0001	6	10
Dr_Monoamine_GPCRs_WP1389_81184	0.0005	9	29
Dr_G_Protein_Signaling_Pathways_WP1371_71520	0.0007	16	75
Dr_Hypothetical_Network_for_Drug_Addiction_WP1333_68646	0.001	8	24
Dr_Striated_Muscle_Contraction_WP1316_68687	0.01	7	29
Dr_Biogenic_Amine_Synthesis_WP154_77401	0.02	4	11
*Significantly activated pathways in upregulated genes*			
Dr_Heme_Biosynthesis_WP1314_71976	0.001	4	8
Dr_Nodal_Signaling_Pathway_WP341_71978	0.004	13	101
Dr_FGF_signaling_pathway_WP152_84645	0.006	17	132
Dr_ERK1_-_ERK2_MAPK_cascade_WP402_71500	0.008	18	153
Dr_TGF_Beta_Signaling_Pathway_WP1370_68675	0.01	7	43
Dr_Apoptosis_WP1351_71509	0.02	9	60
Dr_Apoptosis_Modulation_by_HSP70_WP1392_77556	0.02	4	16
Dr_Toll-like_receptor_signaling_pathway_WP1384_77485	0.02	10	70
Dr_Cholesterol_Biosynthesis_WP1387_77424	0.02	4	16
Dr_FAS_pathway_and_Stress_induction_of_HSP_regulation_WP511_68680	0.03	6	34
Dr_Adipogenesis_WP1331_85024	0.04	12	99

**Figure 6:**
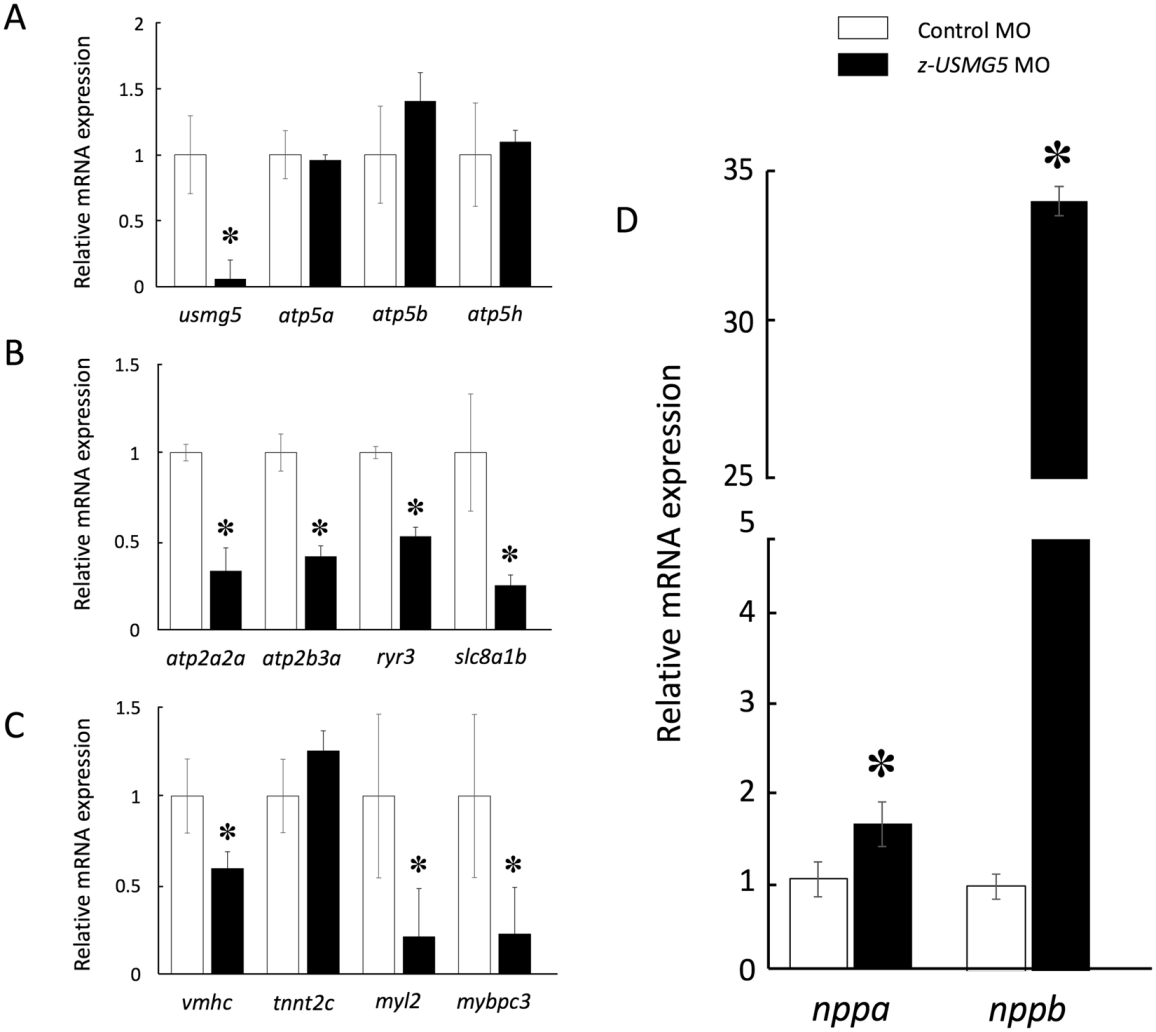
Quantitative real-time PCR confirmed that the expression level of *z-usmg5* was significantly downregulated in *z-usmg5* MO-injected embryos. However, the expression levels of other components of ATP synthase such as *atp5a*, *atp5b*, and *atp5h* were not significantly changed (**A**). The expression levels of major factors in the calcium signalling pathway, such as *atp2a2a*, *atp2b3a*, *ryr3*, and *slc8a1b*, were significantly downregulated (**B**) and cardiac-specific sarcomere-associated genes, such as *vmhc*, *mybpc3*, and *myl2*, were downregulated (**C**) in *z-usmg5* MO embryos compared to in control MO embryos. Furthermore, the expression levels of the natriuretic peptide a (*nppa*) and b (*nppb*) were significantly upregulated (**D**) in *z-usmg5* MO embryos compared to in control MO embryos. *P < 0.05 compared to the respective control. Each bar indicates the mean ± SD of three independent experiments. White and black bars indicate control MO embryos and *z-usmg5* MO embryos, respectively. The results are representative of three independent experiments. Statistical analyses were performed using Student’s *t* test.

Quantitative real-time PCR confirmed that *z-usmg5* knockdown did not affect other components of ATP synthase such as *atp5a*, *atp5b*, and *atp5h* (Fig. 6A). Additionally, we confirmed that the expression levels of major factors involved in calcium signalling including *atp2a2*, *slc8a1b*, *ryr3*, and *atp2b3* were significantly downregulated (Fig. 6B). The expression levels of cardiac-specific sarcomere genes including *vmhc* (cardiac myosin heavy chain), *myl2* (cardiac myosin light chain), and *mybpc3* (cardiac myosin binding protein c) were significantly downregulated in *z-usmg5* MO embryos compared to the control MO embryos (Fig. 6C). Furthermore, the expression levels of natriuretic peptides, such as *nppa* (NM_198800) and *nppb* (NM_001327776), were significantly upregulated in *z-usmg5* MO embryos compared to in control MO embryos (Fig. 6D).

## Discussion

In this study, we demonstrated that *z-usmg5* MO-injected zebrafish embryos showed (1) reduced ventricular contraction and pericardial sac enlargement, (2) downregulated expression levels of major factors in the calcium signalling pathway, and (3) gene expression fluctuations of natriuretic peptides and cardiac sarcomere-associated peptides. These findings indicate that *z-usmg5* knockdown can cause heart failure phenotypes associated with DCM in zebrafish embryos.

DAPIT, encoded by *USMG5*, consists of 58 amino acids^12^ and is known to be a F_o_ subunit component of mitochondrial ATP synthase^13,14^. Although whether DAPIT contributes to ATP production and maintaining ATP concentrations in cardiomyocytes is unclear, several studies have revealed the functions of this protein. Ohsakaya *et al*. showed that mitochondrial ATP production was significantly reduced in DAPIT-depleted HeLa cells^11^, indicating that DAPIT plays a critical role in ATP production by mitochondrial ATP synthase.

ATP synthase is the major generator of ATP in the mitochondrial respiratory chain and maintains ATP concentrations in cardiomyocytes^7^. As the heart requires ATP synthesis than any other organ, cardiac dysfunction in *z-usmg5* knockdown zebrafish embryos may result from mitochondrial ATP synthase dysfunction and subsequent intracellular ATP depletion (Suppl. Figure S4)^8^. Indeed, our data demonstrate that GO terms related to ATP driving intracellular ion transfer, such as ATPase activity, were significantly downregulated in *z-usmg5* knockdown zebrafish embryos (Table 1), supporting this hypothesis. Additionally, we demonstrated that factors in the calcium signalling pathway, a major pathway activated in the failing heart, were significantly downregulated in *z-usmg5* knockdown embryos (Fig. 5). ATP synthase dysfunction resulted in excessive reactive oxygen species (ROS) accumulation in the cytosol, which can cause calcium overload in cardiomyocytes (Fig. 5)^15^^,^^16^. Previous studies demonstrated that intracellular accumulation of ROS leads to the deterioration of the calcium regulator pumps in the sarcoplasmic reticulum (SR) membrane, such as RyR and SERCA, and in the cell membrane, such as NCX^17–20^. Subsequently, calcium overload may lead to cardiomyocyte apoptosis^21^ and myocardial fibrosis^22^, resulting in contractile dysfunction of cardiomyocytes (Suppl. Figure S4)^23^^,^^24^. Further, upregulation of the TGF-β signalling pathway (Table 2) and foetal cardiac genes (Fig. 6D) in *z-usmg5* knockdown embryos may contribute to the development of pathological cardiac remodelling^25^^,^^26^. As the activity of the mitochondrial respiratory chain including the ATP synthase is reduced in the failing myocardium^8,27^, the intervention of ATP synthase-interacting proteins modulating ATP synthase function may be a novel therapeutic target for heart failure in DCM patients. To date, cyclophilin D^28^, ATPase inhibitor factor 1^29^, and protein kinase C delta^30^ have been reported to interact with and modulate ATP synthase function. For DAPIT, Kontro *et al*. showed that overexpression of DAPIT saturated the respiratory chain by decreasing H^+^-ATP synthase activity, leading to increased mitochondrial membrane potential and superoxide levels in human embryonic kidney 293 cells^14^. Taken together with our results, in which DCM phenotypes were detected in *z-usmg5* knockdown zebrafish, these observations reported by Kontro *et al*. indicate that the modulation of DAPIT may be important for maintaining ATP synthase activity and mitochondrial respiratory function and thus improving the cardiac function in heart failure patients. Indeed, co-injection of wild-type *z-usmg5* mRNA with *z-usmg5* MO into the embryos rescued the DCM phenotypes induced by *z-usmg5* MO alone (Fig. 3), suggesting that modulation of *USMG5* mRNA levels can improve cardiac function. Further, expression levels of *USMG5* were generally positively correlated with the severity of heart failure in NICM patients (Fig. 1), indicating that DAPIT expression is compensatory to preserve ATP production during the process of heart failure progression. Together, these findings suggest that DAPIT plays a crucial role in preserving ATP production in the failing heart and thus is a novel target molecule for DCM treatment.

There are several limitations to our study. First, our microarray data was not validated at the protein level by western blot analysis. However, we confirmed the major results of microarray analysis by quantitative real-time PCR. Second, DCM phenotypes in *z-usmg5* knockdown zebrafish embryos should be carefully interpreted because generalized DAPIT knockdown in zebrafish may increase myocardial stress to meet the oxygen demand of peripheral tissues. However, systemic oxygen demand may not be significantly increased in *z-usmg5* knockdown zebrafish embryos compared to in control zebrafish embryos because heart rates were similar between the 2 groups (Suppl. Figure S3). Third, although we suggest that *z-usmg5* knockdown can induce intracellular ATP depletion leading to the heart failure phenotype in zebrafish embryos, further investigations are needed to confirm our results and reveal the role of DAPIT in mitochondrial function. Finally, the statistical evaluations of *NPPA* and *USMG5* expression levels between human control subjects and NICM patients were difficult because of the small sample size (control = 2). Additional study will be necessary to confirm these differences.

Our data demonstrate that *z-usmg5* knockdown induced phenotypes recapitulating heart failure in human with reduced ventricular contraction in zebrafish embryos. DAPIT, a component of ATP synthase, may play a crucial role in the pathogenesis of heart failure associated with DCM and thus could be a therapeutic target for heart failure.

## Methods

### Ethics

This study complied with the Declaration of Helsinki. All zebrafish experimental protocols were approved by the Animal Care and Use Committee of Kanazawa University. In addition, all the experimental protocols used in this study were approved by the Bioethical Committee of Medical Researches of Kanazawa University.

### Microarray analysis using human failed myocardium

Eight RNA samples extracted from human failed myocardium and two samples of normal control subjects were used. Failed myocardium samples were obtained from patients with non-ischemic cardiomyopathy (NICM) who underwent cardiac reconstructive surgery, such as a Batista or Dor procedure, after obtaining written informed consent^31^. The use of myocardial samples was approved by the Bioethical Committee of Medical Researches of the Osaka University (Suita, Japan). Gene expression levels were evaluated using a HG-U95 Affymetrix GeneChip. All expression data were normalized by global scaling and were analysed using GeneSpring software (Agilent Technologies, Santa Clara, CA, USA)^31^.

### Animals

All zebrafish experiments were performed in accordance with the Guide for the Care and Use of Laboratory Animals (National Institutes of Health Publication, 8th Edition, 2011). Zebrafish were raised and maintained at 28 °C on a 14:10 h light-dark cycle. We used the hspGFF3A strain of zebrafish, which expresses green fluorescent protein in the heart^32^, to analyse the size and function of the cardiac ventricle.

### *In vitro* synthesis of zebrafish usmg5 mRNA

The cDNA of the wild-type zebrafish *usmg5* orthologue, *z-usmg5* (NM_001200033), was subcloned into a PCS2P + plasmid expression vector. Capped mRNA was synthesized using mMESSAGE mMACHINE SP6 Transcription Kit (Thermo Scientific, Waltham, MA, USA) according to the manufacturer’s protocol.

### Design of z-usmg5 morpholino oligonucleotides and injection

The morpholino (MO) oligonucleotides were synthesized using Gene-Tools (Philomath, OR, USA). To knockdown *z-usmg5*, we designed a splice site blocking MO to disrupt the translation of *z-usmg5*. The sequence of the acceptor site MO was 5′-GTATGCAATCTGTTAATAAAGGAGA-3′ and that of the donor site MO was 5′-TAATGTCGACTTACATTCCTCCTGC-3′. We also designed control MOs with 5-base pair mismatches corresponding to each of the *z-usmg5* MOs. The control MO sequences for the donor and acceptor MOs were 5′-GTATaCAATaTaTTAATAAAaGAaA-3′ and 5′-TAATaTCGAaTTAaATTCaTCCTaC-3′, respectively. Both *z-usmg5* MOs (2.5 ng for the acceptor and donor site MO) or equal amounts of control MOs were injected into the hspGFF3A zebrafish strain embryos at the 1-cell stage using a microinjector. For rescue experiments, we co-injected synthesized wild-type *z-usmg5* mRNA (400 pg) with *z-usmg5* MO into the embryos.

### Quantification of cardiac morphology and function in zebrafish embryos

Zebrafish hearts at 72 h post fertilization (hpf) were recorded using a Leica digital camera (DFC 310 for colour images and DFC 365 FX for fluorescence images) on a fluorescence stereomicroscope M205A (Leica, Wetzlar, Germany). Cardiac morphologies such as ventricular area, atrial size, and pericardial sac area were quantified with LAS AF software (version 3.1.0; Leica). The end-diastolic ventricular dimension was measured at its largest point. Ventricular fractional shortening was evaluated with recorded movies converted to M-mode images using the original software^31,33^.

### RNA extraction, cDNA preparation, and microarray analysis of zebrafish embryos

Total RNA was isolated from the whole bodies of 72 hpf zebrafish embryos homogenized in 500 μL of RNA BEE reagent (Tel-Test, Friendswood, CA, USA). Subsequently, cDNA was synthesized from extracted RNA using the One-Step reverse transcription PCR Kit (QIAGEN, Hilden, Germany) according to the manufacturer’s protocol. The integrity and concentration of extracted RNA and cDNA were evaluated using the Nano Drop 2000 (Thermo Scientific). The aliquots were stored at −80 °C. We performed microarray analyses to evaluate the influence of *z-usmg5* knockdown on gene expression of zebrafish embryos. RNA samples isolated from wild-type embryos, control MO, and *z-usmg5* MO-injected embryos were compared. Gene expression was evaluated using the GeneChip Zebrafish Genome array containing the probes for 15,509 genes provided by Affymetrix (Santa Clara, CA, USA). Microarray data were analysed using GeneSpring GX software (version 12.1; Agilent Technologies). Gene Ontology (GO) annotation of genes was obtained from the NCBI gene database. Pathway enrichment analysis was performed using the Gene Spring GX software importing WikiPathways (http://www.wikipathways.org)^34^.

### Quantitative real-time PCR analysis

The primer sequences of *z-usmg5* were designed using Primer3Plus (primer3plus.com) and additional primer sequences were taken from the literature^35–38^ (Suppl. Table S2). Quantitative real-time PCR was performed in a total volume of 25 μL, comprised of 12.5 μL of SYBR Green Master Mix (Thermo Fisher Scientific), 50 ng of cDNA, and 200 nM of each primer using an Stratagene Mx3000P qPCR system (Agilent Technologies). Duplicate reactions were performed for each cDNA sample. Forty amplification cycles were performed, with each cycle consisting of 95 °C for 30 s followed by 60 °C for 1 min. Amplification and dissociation curves generated by the MxPro QPCR software (version 4.10; Agilent Technologies) were used for gene expression analysis. The relative quantification of expression of each gene was normalized to the gene expression of *gapdh*.

### Statistics

All analyses were performed using commercially available software (JMP 9.0, SAS Institute, Cary, NC, USA). Data are presented as the mean ± SD. Demographic data were expressed as continuous and categorical variables. Comparisons between continuous variables were performed with the Student’s *t* test or Mann-Whitney U test. Categorical variables were compared using the chi-square test. P values < 0.05 were considered statistically significant.

## Electronic supplementary material

Supplementary information
