# Author Correction: Heart Failure Phenotypes Induced by Knockdown of DAPIT in Zebrafish: A New Insight into Mechanism of Dilated Cardiomyopathy

**DOI:** 10.1038/s41598-018-26012-4

**Published:** 2018-05-14

**Authors:** Yoji Nagata, Masakazu Yamagishi, Tetsuo Konno, Chiaki Nakanishi, Yoshihiro Asano, Shin Ito, Yuri Nakajima, Osamu Seguchi, Noboru Fujino, Masa-aki Kawashiri, Seiji Takashima, Masafumi Kitakaze, Kenshi Hayashi

**Affiliations:** 1 Division of Cardiovascular Medicine, Kanazawa University Graduate School of Medicine, Kanazawa, Japan; 2 Department of Cardiovascular Medicine, Osaka University Graduate School of Medicine, Suita, Japan; 3 Department of Clinical Research and Development, National Cerebral and Cardiovascular Center, Suita, Japan; 4 Department of Cell Biology, National Cerebral and Cardiovascular Center, Suita, Japan; 5 Department of Transplantation, National Cerebral and Cardiovascular Center, Suita, Japan; 6 Department of Medical Biochemistry, Osaka University Graduate School of Medicine, Suita, Japan

Correction to: *Scientific Reports* 10.1038/s41598-017-17572-y, published online 12 December 2017

The original version of this Article contained an error in the title of the paper, where the word “Heart” was incorrectly given as “Heat”. This has now been corrected in the PDF and HTML versions of the Article, and in the accompanying Supplementary Information file.

