# Supplementary material for: Heart Failure Phenotypes Induced by Knockdown of DAPIT in Zebrafish: A New Insight into Mechanism of Dilated Cardiomyopathy
Source: Sci Rep. 2017 Dec 12;7:17417. doi: 10.1038/s41598-017-17572-y (PMC5727169; doi:10.1038/s41598-017-17572-y)
Supplement: Supplementary file 1 — Supplementary information [file 41598_2017_17572_MOESM1_ESM.pdf]

Title: Heart Failure Phenotypes Induced by Knockdown of DAPIT in Zebrafish: A New Insight into Mechanism of Dilated Cardiomyopathy

Short title: DAPIT and Heart Failure

Yoji Nagata, M.D.[1], Masakazu Yamagishi, M.D., Ph.D.[1,*], Tetsuo Konno, M.D., Ph.D.[1], Chiaki Nakanishi M.D., Ph.D.[1], Yoshihiro Asano, M.D., Ph.D.[2], Shin Ito, M.D., Ph.D.[3], Yuri Nakajima, M.D.[4], Osamu Seguchi, M.D., Ph.D.[5], Noboru Fujino, M.D., Ph.D.[1], Masa-aki Kawashiri, M.D., Ph.D.[1], Seiji Takashima, M.D., Ph.D.[6], Masafumi Kitakaze, M.D., Ph.D.[3], Kenshi Hayashi, M.D., Ph.D.[1]

[1] Division of Cardiovascular Medicine, Kanazawa University Graduate School of Medicine, Kanazawa, Japan

[2] Department of Cardiovascular Medicine, Osaka University Graduate School of Medicine, Suita, Japan

[3] Department of Clinical Research and Development, National Cerebral and Cardiovascular Center, Suita, Japan

[4] Department of Cell Biology, National Cerebral and Cardiovascular Center, Suita, Japan

[5] Department of Transplantation, National Cerebral and Cardiovascular Center, Suita, Japan

[6] Department of Medical Biochemistry, Osaka University Graduate School of Medicine, Suita, Japan

Suppl. Figure S1

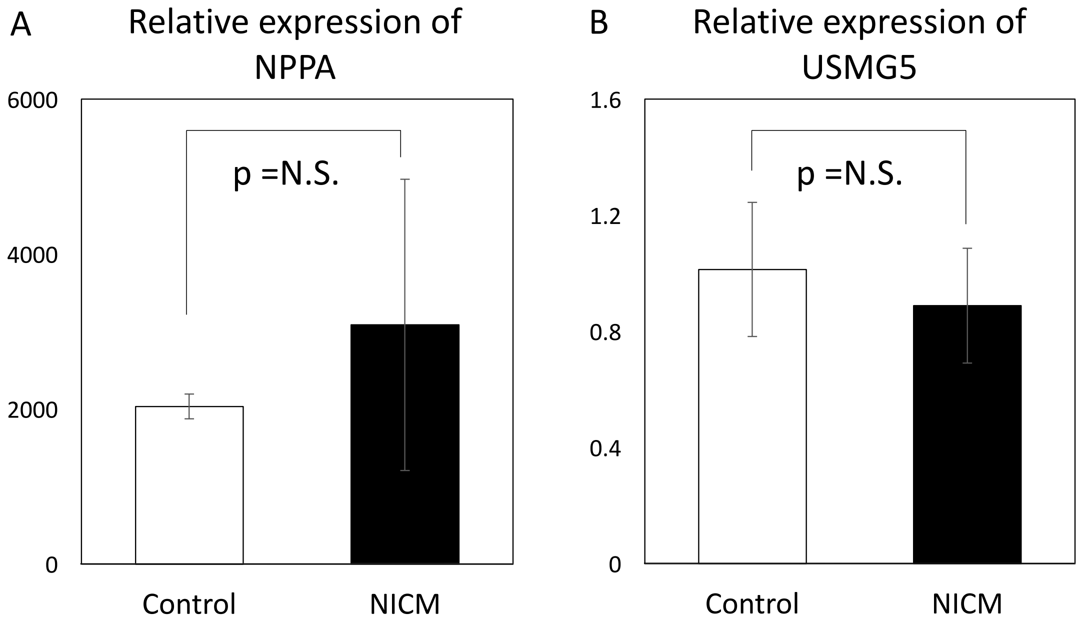

Supplementary Figure S1. There were no differences in the relative expression levels of (A) *NPPA* (2032 ± 159 vs. 3086 ± 1878, $p = 0.60$) and (B) *USMG5* (1.01 ± 0.23 vs. 0.88 ± 0.19, $p = 0.29$) in the myocardium between control subjects (n = 2) and NICM patients (n = 8), respectively. Statistical analyses were performed using Mann-Whitney U test. N.S. indicated not significant ($p > 0.05$), *NPPA*: natriuretic peptide precursor A, NICM: non-ischemic cardiomyopathy.

Suppl. Figure S2

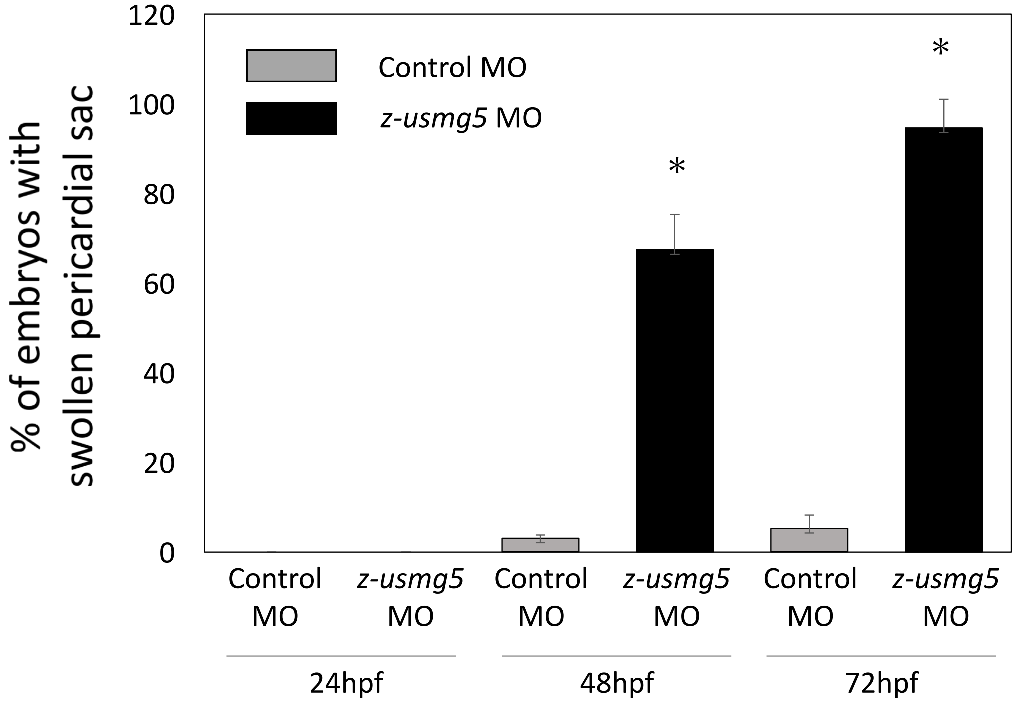

Supplementary Figure S2. Percentage of embryos with swollen pericardial sac (>2SD from wild-type embryos) at each of 24, 48, and 72 hpf. Compared to control MO-injected embryos, the percentage of embryos with swollen pericardial sac were significantly greater at 48 and 72 hpf in *z-usmg5* MO-injected embryos. * indicates $p < 0.0001$ compared to control-MO injected embryos. The results are representative of three independent experiments. Statistical analyses were performed using Mann-Whitney U test.

Suppl. Figure S3

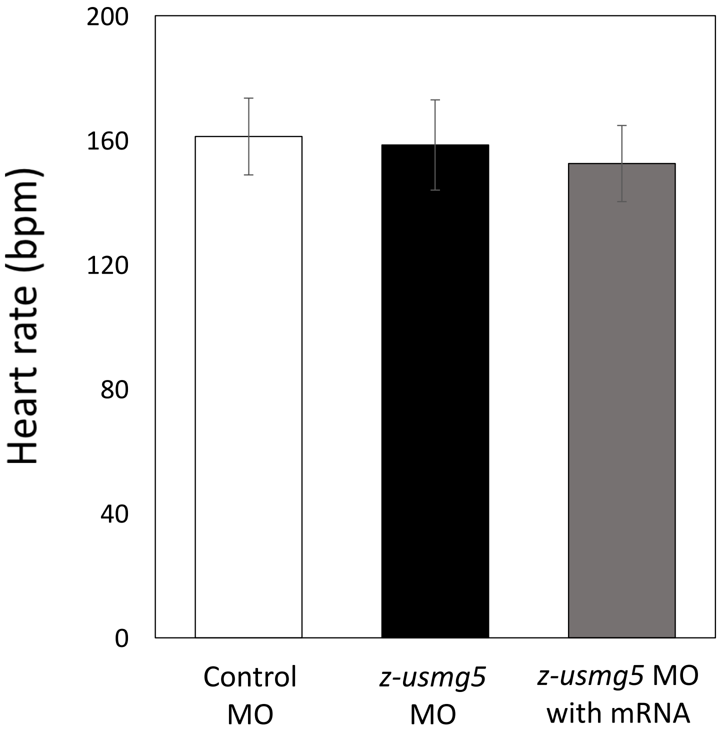

Supplementary Figure S3. Quantitative measurements of heart rates in zebrafish embryos injected with control MO (control MO group, n = 21), *z-usmg5* MO (*z-usmg5* MO group, n = 19) and *z-usmg5* MO with its wild-type mRNA (*z-usmg5* MO with mRNA group, n = 20). There were no differences in heart rates between control MO (161 ± 12 bpm), *z-usmg5* MO (158 ± 14 bpm), and *z-usmg5* MO with its wild-type mRNA (152 ± 12 bpm) groups. Statistical analyses were performed using Mann-Whitney U test. bpm: beats per minute.

Suppl. Figure S4

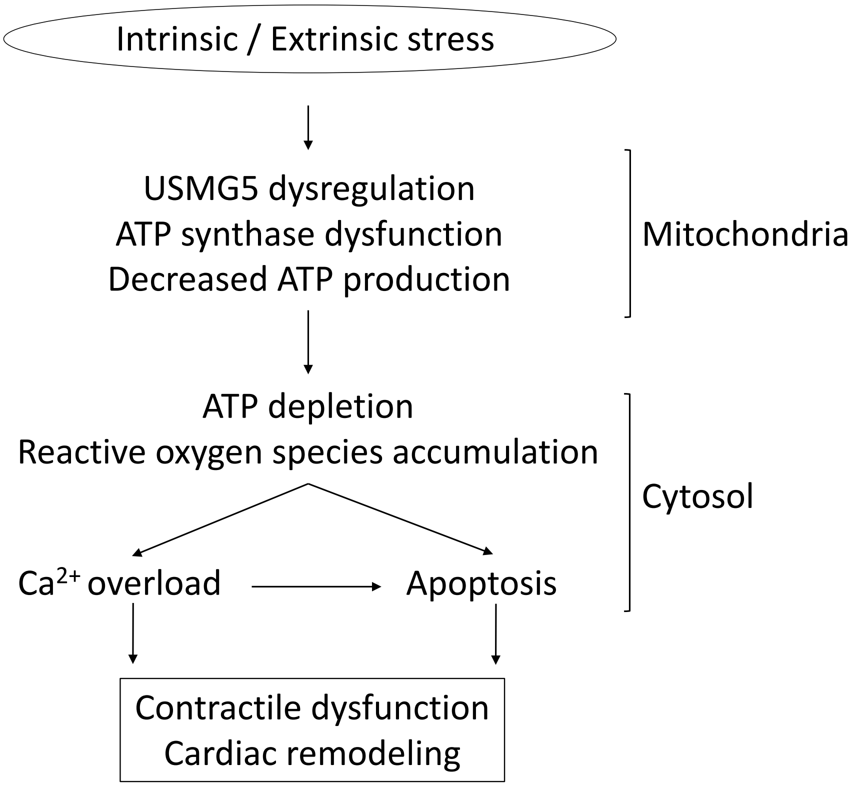

Supplementary Figure S4. *USMG5* knockdown leads to myocardial dysfunction, resulting in progression of heart failure. ATP synthase dysfunction related to intrinsic *USMG5* dysregulation induced by cellular stress such as ischemia, hypoxia, pressure overload, and DNA damage could reduce mitochondrial ATP production and lead to intracellular ATP depletion. Subsequently, intracellular reactive oxygen species accumulation induces alterations in calcium handling and the activation of the apoptosis signalling pathway related to myocardial contractile dysfunction and cardiac remodelling resembling DCM.

Supplementary Table S1. Quantitative measurements of cardiac dimensions and functions in wild-type and control-MO-injected embryos.

	Wild-type	Control-MO	p
Pericardial sac area (μm^2)	33533 ± 5069	32962 ± 6295	0.67
Atrial area (μm^2)	8406 ± 1145	9052 ± 1361	0.10
Ventricular diastolic diameter (μm)	93.7 ± 9.1	95.8 ± 10.1	0.46
Fractional shortening (%)	20.7 ± 5.8	22.0 ± 6.0	0.47

There were no differences in pericardial sac area, atrial area, ventricular diastolic diameter, or fractional shortening between wild-type (n = 21) and control-MO injected (n = 21) embryos. Statistical analyses were performed using Mann-Whitney U test.

Supplementary Table S2. Primers list for quantitative polymerase chain reaction of zebrafish.

	Forward	Reverse
atp2a2a	ATCGGTAAGATCCGTGATGA	CACGGCGATCTTGAAGTAGT
atp2b3a	TTGCTGGTACAGATGTGGC	GGAGGAGAGTGAAGCGGAG
atp5a	TCAGAGACAACGGCAAACAC	GTGGGAATGTAAGCGGACAC
atp5b	GTCACAGGAATCAAGGTCGTA	GCTCCATAATCAACACAGTCT
atp5h	TGCCATCTCAGCAAAACTTG	CACAGGCTCAGGAACAGTCA
gapdh	CCACCCATGGAAAGTACAAG	CTCTCTTTGCACCACCCTTA
myl2	TGCATAGATCAGAACCGGGA	ATTATAATTTCACTAGATGT
mybpc3	TGCCGGAGCCTATCGCTGTGA	ATGAACAGCTCGCTGGCCCGT
nppa	GATGTACAAGCGCACACGTT	TCTGATGCCTCTTCTGTTGC
nppb	CATGGGTGTTTTAAAGTTTCTCC	CTTCAATATTTGCCGCCTTTAC
ryr3	AAGGACAGTGGGATCGTTTG	AAAGTCCAGCGCACTCTTGT
slc8a1b	AGAGACGAGGAGAAGGAGGT	GCACGAAAGCAAAGAGAACT
tnnt2c	GACCGAACGTGAGAAGAAGA	AGGACTTCCTGGTGGTTTTC
vmhc	TCAGATGGCAGAGTTTGGAG	GCTTCCTTTACAGTTACAGTCTTTC
